# Impact of *Oreochromis niloticus* (Linnaeus, 1758) (Pisces: Cichlidae) invasion on taxonomic and functional diversity of native fish species in the upper Kabompo River, northwest of Zambia

**DOI:** 10.1002/ece3.8031

**Published:** 2021-08-19

**Authors:** Arthertone Jere, Wilson W. L. Jere, Austin Mtethiwa, Daud Kassam

**Affiliations:** ^1^ Department of Aquaculture and Fisheries Science Africa Centre of Excellence in Aquaculture and Fisheries (AquaFish ACE) Faculty of Natural Resources Lilongwe University of Agriculture and Natural Resources Lilongwe Malawi; ^2^ Department of Fisheries, Extension services Ministry of Fisheries and Livestock Solwezi Zambia

**Keywords:** biological invasion, ecosystem, exotic species, functional trait, Zambia

## Abstract

Invasive alien species have been revealed to drastically alter the structure of native communities; however, there is scarce information on whether taxonomic and functional spaces occupied by native species are equally filled by exotic species. We investigated the diversity of native species to understand the impact of exotic *Oreochromis niloticus* in the upper Kabompo River, northwest of Zambia using taxonomic and functional diversity indices. To achieve this, two tests were performed (Test 1, compared natives in invaded and uninvaded sections; Test 2, compared natives in invaded section). A total of 17 species were collected for functional diversity computation, out of which fourteen (14) functional trait measurements linked to feeding, locomotion, and life history strategy were taken. Findings revealed that taxonomic and functional diversity values changed with invasion in both tests. Taxonomic diversity was 15% more in invaded than uninvaded sections in Test 1 and was not consistent across sampling points of invaded section in Test 2. Invaded areas were taxonomically less diverse, but functionally diverse in both tests. The analysis of similarity and nonmetric multidimensional scaling revealed no difference in Bray–Curtis similarity assemblages in both tests. Our findings revealed that exotic species more often occupy unfilled gaps in the communities often occupied by the native species; this is achieved by occupying functional spaces. Overall, changes in taxonomic and functional diversity of native species documented here partially confirmed impacts of *O*. *niloticus* invasion. Therefore, we recommend a multifaceted approach to assess cumulative impacts of invasion on native species.

## Graphical abstract

Nile tilapia invasion.

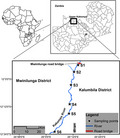

## 1 INTRODUCTION

Invasion by exotic species has become a significant danger to biodiversity of communities and functioning of freshwater ecosystem worldwide (Hill et al., 2020; Weyl et al., 2020). Biological invasion impact negatively on the capacity of ecosystems to deliver goods and services and in some cases severely threaten human livelihood (Cumming & Child, 2009; Ellender et al., 2018; van Wilgen et al., 2020; Zengeya et al., 2020) provides an insight in understanding the functioning of ecosystem (Hill et al., 2020; Le Maitre et al., 2020; Mouillot et al., 2013). Investigating the implications and systems of fish invasion remains crucial in quantifying taxonomic and functional similarities among local and exotic species (Cumming & Child, 2009; Mouillot et al., 2013; Mindel et al., 2016; Williams et al., 2016; van Wilgen et al., 2020). Nile tilapia, *Oreochromis niloticus* (Linnaeus, 1758), is the most widely used fish species for aquaculture in Africa, due to their rapid growth rate, general hardiness, ability to efficiently utilize organic wastes, and ease of breeding (Ellender et al., 2018; Weyl et al., 2020; Zengeya et al., [Link], 2020). In Zambia, it was introduced for aquaculture development in the early 1980s (FAO, 2012; Bbole et al., 2014; DoF, 2018). However, predicting the response of native species in the ecosystem to fish species invasion remains a critical ecological question (Jardine et al., 2017; Le Roux et al., 2020; Wilson et al., 2020). Therefore, quantifying the taxonomic and functional diversity related to traits of species is viewed as useful approaches to better understand diversity of species within communities (Carboni et al., 2016; Le Maitre et al., 2020).

The study reviewed two theoretical predictions that are seemingly contradicting each other have been provided in this study to predict impact of exotic species on native species in ecological communities. Firstly, we reviewed the hypothesis on environmental filtering (Carboni et al., 2016), which states that exotic species must adapt in a related environment as native species. This suggests that species that are related to each other might have a good opportunity to coexist in that particular community. In this study, taxonomic diversity (TD) refers to variety of species, while functional diversity (FD) is the variation of the functional traits of species in a given community (Cumming & Child, 2009; Jardine et al., 2017; Pease et al., 2012). According to this concept, exotic species in biological networks frequently fill a segment of the taxonomic and functional space initially involved by native species (Cumming & Child, 2009; Le Maitre et al., 2020; van Wilgen et al., 2020; Wilson et al., 2020). While Darwin's naturalization hypothesis (Rejmanek, 1996) is a second prediction, which states that exotic species are more likely coexist with native species by stochastic processes (Le Roux et al., 2020; Thuiller et al., 2010; van Wilgen et al., 2020). Presumptions of this speculation demonstrate that specialty separation firmly corresponds with “limiting similarity.” This predicts that specialty hole filling by exotic species is significant to their effective foundation in the ecosystem. Subsequently, this may prompt diversity of native species in invaded communities. The upper Kabompo River is one of the most diverse rivers with so far 61 native species that have been caught during the year 2013, 2015, and 2016 monitoring surveys (DoF, 2018). As such, increase or decrease in taxonomic and functional diversity may be linked to the presence or absence of exotic species in an ecosystem (Cumming & Child, 2009; Hulme and Bernard‐Vertier, 2017; Jardine et al., 2017; Mindel et al., 2016; Zengeya et al., 2020).

There are two outstanding factors that seem to be responsible for the differences in both environmental filtering and limiting similarity hypotheses. On the one hand, the two theories are scale subordinate (Cumming & Child, 2009; Gotzenberger et al., 2016; Ellender et al., 2018; Weyl et al., 2020); subsequently, ecological shifting is relied upon to be the main factor driving assemblage across habitats. In that capacity, specialty sharing and gap filling are relied upon to be more significant for concurrence species at community scale or in a given habitat. Therefore, environmental filtering assessment requires the examination of taxonomic diversity and species function across habitats. Further, we expect to find aggregation in the assembly of native species regarding taxonomic and functional diversity in the communities. According to Lososova et al. (2015), closely related species have shown to occupy similar habitats; thus, exotic species with greater similarity to native species are likely coexist in invaded communities (Cumming & Child, 2009; van Wilgen et al., 2020; Wilson et al., 2020). On the other hand, observational studies have been the main method that has been used in evaluating these hypotheses.

To comprehensively understand the impacts of invasion, we investigated the influence of *O*. *niloticus* on the TD and FD of resident species in the upper Kabompo River, one of the largest and the deepest rivers in Zambia. To our knowledge, this represents the first investigation of TD and FD relationship of tropical freshwater fishes in the river. The patterns of fish assemblage structure may not conform to expectations based on studies in temperate regions owing to differences in zoogeography and climate. However, the consideration of TD in this system is significant as (1) the integral data of TD and FD can assist us with bettering foresee the cycle of fish invasion and (2) it may uncover design not perceptible simply by the attributes considered to process FD (Azua et al., 2017; Ordonez, 2014). Further, while it is becoming increasingly clear that TD independently cannot be used, but as a precursor to FD (Le Roux et al., 2020; Mouillot et al., 2013; van Wilgen et al., 2020). Taxonomic diversity assessment alongside FD allows for broader predictions in cases where information on traits is missing (Lososova et al., 2015; van Wilgen et al., 2020).

Based on the expectation of the previous studies of fish diversity across the Kabompo River (AES, 2014; Bok & Bills, 2012; DoF, 2018), we predicted that TD and FD would be different in the community invaded than the uninvaded areas of the upper Kabompo River and this would reflect the possible effect of invasion across the river. Also, we solely expected invasion would influence taxonomic and functional structure across the river segments than other environmental related conditions. This is because, the environmental parameters such as pH, water temperature, conductivity, salinity, and water flow rates showed similarity in all sampling points of the upper Kabompo River. However, we still anticipated that exceptions to this trend might occur where river habitat conditions differ from general expectations based on longitudinal position such fine substrates and slightly warm temperatures in certain unsampled habitats which had tributary connection in different points of the river. In accordance with the two aforementioned theoretical predictions, we expected smaller, flashy headwater of the sampled points of the river to be characterized by fishes with similar traits and possible fish species diversity related to such slight variations in environmental conditions. Generally, we expected that relationship between the native fish species cross the habitats of the study area would reflect the influence of invasion at both local and landscape spatial scales.

This knowledge of how species taxonomy and functional traits of resident fishes respond to invasion will increase our understanding on impacts of *O*. *niloticus*. Also, it will help us to drawing prudent fishery's management plans in fisheries that are invaded or potentially threatened water bodies.

## 2 MATERIALS AND METHODS

### 2.1 Description of the study area

**FIGURE 1:**
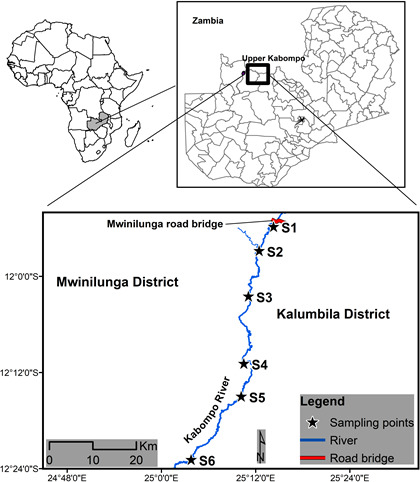
Map of the study area—upper Kabompo River

The survey was conducted in the upper Kabompo River situated in the Northwestern province of Zambia (Figure 1). The study area stretches ~45 km in length from the source of the Kabompo River (latitude 25.2414 to 25.044156 E and longitude 11.8973 to −12.369120 S, respectively). The upper Kabompo is part of the Kabompo River and is one of the main tributaries of the Zambezi River. It originates at an altitude of approximately 1,500 m above mean sea level (amsl) in the highlands which form the watershed between the Zambezi and Congo rivers. The river flows in a general south‐westerly direction from the highlands through flatter areas before entering the deep narrow valley of the Kabompo Gorge at Wushingi Hills. Within the Kabompo Gorge, the river course disappears below rocks and boulders (creating a natural barrier during dry season) and flows underground for ~1.5 km where it reemerges below a major drop‐off in the river gorge ~40–50 m in elevation. The upper Kabompo River channel including floodplain during peak rain season is up to 1km wide in this upper section of the river, and numerous tributaries join the Kabompo River between the river and the villages. The average water temperature is about 25°C across the study area. The study site was chosen because it is the most active area for artisanal fishing activities and has been invaded by *O*. *niloticus* (Bok & Bills, 2012; AES, 2014). The fishery also provides a livelihood to the surrounding local communities and a source of brood fish for Aquaculture Breeding Programme for the Department of Fisheries (DoF, 2018).

### 2.2 Sampling procedure

During the survey, 30 km of the river stretch was sampled, which was divided into two main sections, each covering 15 Km stretch. One section is invaded by *O*. *niloticus*, while the other section is uninvaded. The two sections were separated by a unique natural water fall and created a conductive environment to perform good comparison in our study. Both the invaded and uninvaded sections are each located 5.4 km away from the unique natural water fall. At each sampled section, a total of three (3) sampling points were selected with a river distance of about 4.5 km apart from each other. Each sampling point had a total of 200 m stretch covered encompassing all the available five (5) microhabitat type (runs, riffles, vegetative thicket point, open pool (near fisheries landing area) and the tributaries. Sampling of the fish was conducted in the entire 5 selected aforementioned habitat present in each sampling point. Therefore, a total of six sampling points with 30 microhabitats were sampled in both the invaded and uninvaded sections of the upper Kabompo River from December 2019 to February 2020.

**FIGURE 2:**
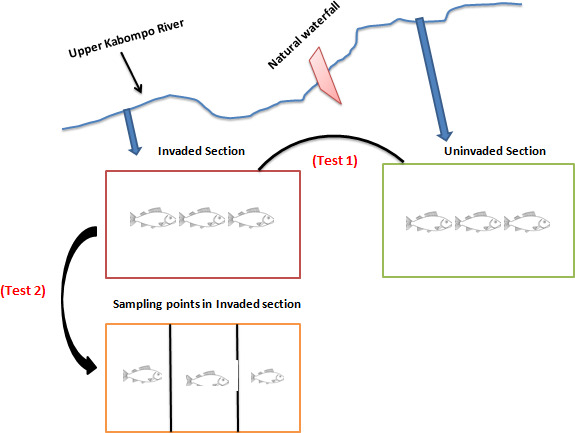
Conceptual model of the two tests comparing taxonomic and functional diversity indices of native species in uninvaded versus invaded sections (Test 1) and native species in sampling points within the invaded section, with high and low presence of exotic species (Test 2) of the upper Kabompo River

Bok and Bills (2012) and DoF (2018) showed that the study area has a greater similarity in native fish species present, rainfall pattern, environmental condition and general water flow regime despite the unique natural barrier that separates them. Due to the proximity of the two sections, it made the two sections of the river easier to compare. The comparison strategy developed here to understand the impact of invasion was a modification from the one proposed by Loiola et al. (2018). This was because the latter comparison was used to compare invasion by terrestrial plants, while here we compared fish invasion. We compared invaded and uninvaded sections to comprehensively investigate the impact of invasion at broader scale (Test 1, Figure 2). As such, TD and FD comparisons between the invaded and uninvaded sections were performed to overall understand the influence of *O*. *niloticus*. Secondly, we compared the high or low presence of the *O*. *niloticus* in the sampling points of the invaded section (Test 2, Figure 2).

### 2.3 Fish sampling

We employed a Fisheries independent (FI) sampling methods to collect fish by using gill nets ranging from 2.5 to 8 cm mesh size and 3 m deep and 30 m longs; two double‐ended fyke nets made from 20 mm stretched multifilament netting with 75 cm D‐ends separated by an 8 m leader; an LR‐24 Electrofisher‐Smith‐Root with 400 watt electrical output; and a beach seine net of 25 m long, and 3 m deep. The fyke and gill nets were deployed parallel to the bank in slow flowing water where boat access was not restricted by fish weirs or fallen tree and inspected in the morning (6 a.m.) and afternoon (17 p.m.) for period of 2 months, and displayed for 24 hr. Electric fishing and seining were conducted during daytime over three days per week within each sampling point. Effort was limited by the amount of suitable habitat for each gear type and restricted to areas where it was deemed safe to operate. Because variations in the morphology of the fishes depend on body size and developmental stages, we selected individuals of each species that corresponded to the adult size class interval. Fish specimens were preserved in 10% formalin and later stored in 75% ethanol before taken to the laboratory for examination. We collected and used 1998 individuals to provide a comprehensive taxonomic description of the native (*n* = 39) and exotic (*n* = 1) fish species in the upper Kabompo River. Thereafter, a total of fourteen (14) traits from at least 17 adult individuals of each species of similar size that were found in both sections were considered for evaluation to minimize variation in functional trait measurements (Table 1). A Vernier caliper was used to take functional trait measurements from the selected specimen at the laboratory. These traits are picked on the grounds that they are notable identified with feeding and life history strategies with attributes like gut length, egg diameter, head length, mouth width, and snout length (Gatz, 1979; Jardine et al., 2017; Olden et al., 2010). In fact, these traits also are potential predictor of the species’ effects on ecosystem functioning (Pease et al., 2012; Stuart‐Smith et al., 2013). Morphological features which are related to locomotion were also taken, which included relative body depth, body width, standard length, dorsal fin length, and caudal peduncle dimensions (Devictor et al., 2010; Gatz, 1979; Winemiller & Rose, 1992).

**TABLE 1:** Functional trait measurement taken from the fish specimen in both the invaded and uninvaded sections of the upper Kabompo River

Trait	Trait code	Trait definition	Functional category
Egg diameter	ED	Mean diameter of mature (fully yolked) oocytes	Life history strategy
Standard length	SL	Length of the fish from the mouth tip to the end of caudal fin	Life history strategy and feeding
Gut length	GL	Length of gut from the being of esophagus to the anus (extended without stretching)	Feeding
Head length	HL	Distance from the tip of the jaw to the posterior edge of the operculum	Feeding
Head depth	HD	Vertical distance from dorsum to ventrum passing through the pupil	Feeding
Oral gape	MW	Vertical distance measured inside of fully open mouth at tallest point	Feeding
Eye diameter	HED	Horizontal distance from eye margin to eye margin	Feeding
Snout length	SNL	Distance from the pupil to the tip of the upper jaw with mouth shut	Feeding
Body width	BW	Maximum horizontal distance from side to side	Locomotion
Caudal peduncle length	CPL	Distance from the posterior proximal margin of the anal fin to the caudal margin of the ultimate vertebra	Locomotion
Dorsal fin length	DFL	Maximum distance from the proximal to distal margin of the dorsal fin (excluding filaments)	Locomotion
Dorsal fin length	BD	Distance from the anterior proximal margin to the posterior proximal margin of the dorsal fin	Locomotion
Caudal fin depth	CPD	Maximum vertical distance across the fully spread caudal fin	Locomotion
Caudal fin length	CFL	Maximum distance from proximal to distal margin of the caudal fin (excluding filaments)	Locomotion

### 2.4 Habitat sampling

Physicochemical variables (temperature, dissolves oxygen, pH, turbidity, and conductivity) were collected and recording daily at each during sampling point. These variables were determined with a YSI model 85 meter except for pH which was measured with a handheld electronic pH meter. Also, the upper Kabompo River is a lotic environment with relatively similar conditions and that the sampling points are in close proximity along the river channel.

### 2.5 Statistical analyses

To quantify biodiversity, two fundamental parts that supplement biological system working administrations were utilized. (1) Taxonomic diversity (TD) that solitary records for the species arrangement and wealth was performed to compare the influence of *O*. *niloticus* invasion as aforementioned in Test 1 (comparing native species in invaded versus uninvaded sections) and Test 2 (comparing the native species in sampling points of the invaded section of the upper Kabompo River) (Figure 2). To do this, specimens were identified to species level and recorded immediately after capture according to the fish species identification guide by Kenzo and Mazingaliwa (2002). Thereafter, (2) functional diversity (FD) that accounts for the ecological traits of species was performed from both the invaded versus uninvaded sections and among native fish species in the invaded section (Test 1, Test 2, Figure 2).

To examine the structuring of TD [gamma(γ, total) diversity] across the invaded and uninvaded sections (Test 1) and sampling points in the invaded section (Test 2) due to invasion was achieved by examining the relative contributions of alpha (α, within sampling points) and beta (β, among sampling points) diversities using the number of fish species observed. A complete randomization of the data through an iterative process was performed using addictive partitioning to test for the presence of pattern across the sampled sites in the upper Kabompo River. The observed fish species richness tested at each site was not significantly different. As such, the computations of TD in both Tests 1 and 2 followed the model used by Freedman et al. (2014).

$$\gamma \left(\;\text{TD}‐\text{total}\right)=\alpha_{1\left(\text{within sampling points}\right)}+\beta_{1\left(\text{among sampling points}\right)}+\beta_{2(\text{among sections})}$$

Simpsons’ index based on the observed data of relative species abundance was calculated and converted into effective numbers of species in the river according to Magurran (1988). Depending on normality and homoscedasticity of TD index mean values, analysis of variance (ANOVA) was run to determine the significant difference in Tests 1 and 2 (Sokal & Rohlf, 1995). Furthermore, we performed nonmetric multidimensional scaling (NMDS) and analysis of similarity (ANOSIM) based on a Bray–Curtis dissimilarity matrix to examine the differences among sites and sections in fish species assemblages as a result of fish invasion in both tests (McCune & Grace, 2002). All these statistical analyses were performed using R software package version 3.6.0 (R Core Team, 2019), package vegan (Oksanen et al., 2019).

To examine the structuring of FD in the sample sites, functional diversity facet computations were performed at each site in both sections. To accommodate for the differences in traits of the species and also traits information that are missing, firstly we performed a Gower distance computation among the species by conducting all pairwise trait dissimilarities (Pavoine et al., 2009). Furthermore, to account for correlations and variation of the traits, a principal coordinate analysis (PCoA) was performed to examine dissimilarity among the species (Laliberte & Legendre, 2010). Therefore, scores that explained 80% of variance on the PCoA axes were chosen for the multivariate FD computation among the species in the sampling area. Thereafter, multidimensional functional diversity indices that reflect; (1) functional evenness (FEve), (2) functional richness (FRic) and (3) functional divergence (FDiv) were performed. These diversity facet measurements are described by REF (Villeger et al., 2008). Functional richness (FRic) influences FD and was achieved by computation of the convex hull volume using the Quick hull algorithm (Barber et al., 1996). The Functional divergence facet of FD quantifies whether higher abundance is close to the volume borders of the habitat (Villeger et al., 2008). The index is constrained between zero and one, where nearing zero represents highly species abundance and one less abundance of species. Functional evenness facet of FD measure describes the evenness of the species distribution in the community. To compute this, the formula used was proposed by REF (Villeger et al., 2008). The FEve index is constrained between zero and one, where zero represents uneven distribution species with similar functional traits in a community, while one indicates evenly distribution of species with similar trait values within a community.

Depending on normality and homoscedasticity of FD indices mean values, analysis of variance (ANOVA) was run to determine the significant difference (Sokal & Rohlf, 1995). Further, Tukey HSD post hoc test was performed to detect differences in their mean values obtained from the invaded and uninvaded sections in Test 1 and among the sampling points in Test 2. Statistical analyses were performed using R software package version 3.6.0 (R Core Team, 2019), package FD (Laliberte & Legendre, 2010).

## 3 RESULTS

**FIGURE 3:**
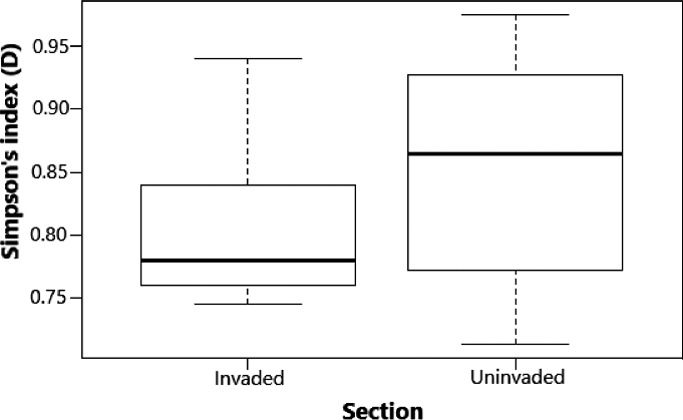
Taxonomic diversity comparison between uninvaded and invaded sections of the upper Kabompo River (Test 1). Whiskers represent ±*SD*, dashed line in box is the median of the dataset, and dash around the box is dataset variation

**FIGURE 4:**
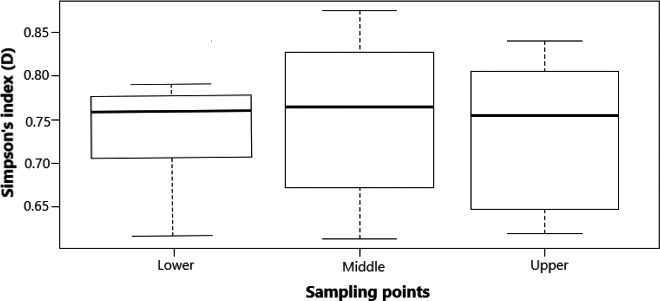
Taxonomic diversity comparison of sampling points within the invaded section of the upper Kabompo River (Test 2). Whiskers represent ±*SD*, dashed line in box is the median of the dataset, and dash around the box is dataset variation

**FIGURE 5:**
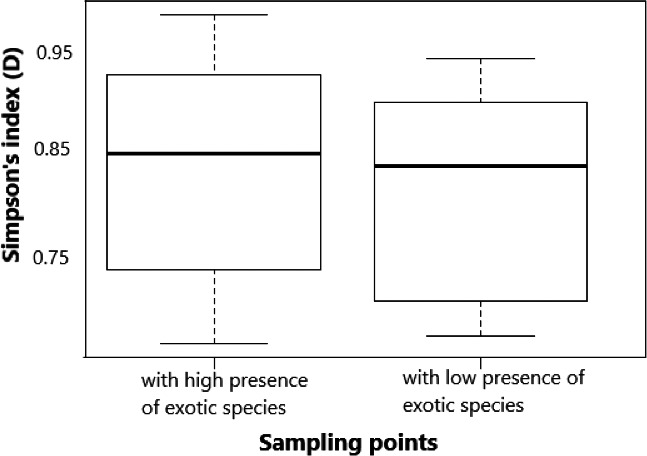
Taxonomic diversity (Test 2) of species within sampling points with high or low exotic species presence in invaded section of the upper Kabompo River. Whiskers represent ±*SD*, dashed line in box is the median of the dataset, and dash around the box is dataset variation

The total number of species collected in invaded section was generally low (47% representing 35 fish species), with increase from lower to upper and then middle sampling points compared with the uninvaded section (53% representing 39 species) with evenly distribution across all the three sampling points (lower, middle, and upper). The mean taxonomic diversity of native species in the invaded section (mean Simpson's index 0.78 ± 0.31 SE) versus uninvaded section (0.86 ± 0.28) of the river stretch in Test 1 was significantly different (*p* = 1.249, significant level 0.05) using the Simpson's index (Figure 3). The index also revealed varying mean taxonomic diversity in the sampling points (lower 0.76 ± 0.04; middle 0.77 ± 0.19; upper 0.76 ± 0.21) and was not significantly different across the sampling points (*p* = 4.339, significant level 0.05) of the invaded section in Test 2 (Figure 4). Further, mean taxonomic diversity of native species in the sampling points with low and high *O*. *niloticus* presence in the invaded section of the river stretch was not significantly different (*p* =0.467, significant level 0.05, with high exotic species 0.85 ± 1.29; with low exotic species 0.84 ± 1.34) (Figure 5). Our findings in Test 1 indicated that TD in the invaded section was lower compared with that of the uninvaded section. In Test 2, the sampling points within the invaded section that had low or high *O*. *niloticus* presence considered were not different, while the mean diversity sampling points of the invaded section did not differ.

**FIGURE 6:**
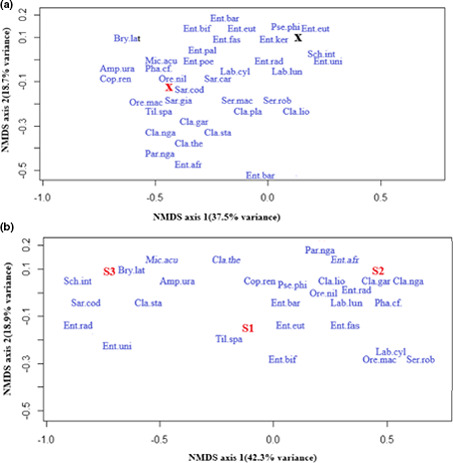
NMDS ordination of Bray–Curtis similarity of a) invaded section (red marked cross) versus uninvaded section (Black marked cross)—Test 1 and b) sampling points (S1–3 marked red) in the invaded section—Test 2 of the upper Kabompo Rivers. *Brycinus lateralis*—Bry.lat, *Amphilius uranoscopus*—Amp.ura, *Pharyngochromis cf. acuticeps*—Phy.acu, *Pseudocrenilabrus philander*—Pse.phi, Sargochromis carlottae—Sar.car, *Serranochromis macrocephalus*—Ser.mac, *Serranochromis robustus jallae*—Ser.rob, *Parauchenoglanis ngamensis*—Par.nga, *Oreochromis macrochir*—Ore. mac, *Clarias ngamensis*—Cla.nga, *Coptodon rendalli*—Cop.ren. *Oreochromis niloticus*—Ore.nil, *Tilapia sparmanii*—Til.spa, *Enteromius afrovernayi*—Ent.afr, *Enteromius barotseensis*—Ent.bar, *Enteromius bifrenatus*—Ent.bif, *Enteromius eutaenia*—Ent.eut, *Enteromius fasciolatus*—Ent.fas, *Enteromius kerstenii*—Ent.ker, Enteromius paludinosus—Ent.pal, *Enteromius poechii*—Ent.poe, *Enteromius radiatus*—Ent.rad, *Enteromius unitaeniatus*—Ent.uni, and *Labeo cylindricus*—Lab.cyl

Differentiation between fish assemblages from the invaded and uninvaded sections of the upper Kabompo River along axes was observed using nonmetric multidimensional scaling based on Bray–Curtis similarity. A total of 54.2% variation was explained (NMDS, Stress 0.16) in abundance of species between two sections, with 35.7% and 18.5% variance in first and second axis, respectively, in Test 1 (Figure 6a). These findings suggested that the presence of *O*. *niloticus* did not confidently expanded fish assemblages of native species in the invaded section. In Test 2, a total of 60.5% of variation was explained (NMDS, Stress 0.12) in abundance of species in sampling points of the invaded section, with 41.7% and 18.9% variance in first and second axes, respectively (Figure 6b). Findings here suggested that the presence of *O*. *niloticus* in the sampling point slightly increased native fish assemblages in the invaded section. The NMDS test in both Tests 1 and 2 was contradictory. Therefore, our results did not clearly show that the presence of *O*. *niloticus* taxonomically influenced the assemblages of native species in the upper Kabompo River.

Analysis of similarity (ANOSIM) between the invaded and uninvaded sections in Test 1 did not reveal any significant difference (Global *R*: 0.12, *p* > 0.05). Fish assemblages in both the invaded and uninvaded sections were similar. In Test 2, ANOSIM among native species in the sampling points with the presence and absence of *O*. *niloticus* was significantly different (Global *R*: 0.68, *p* < 0.05), with fish assemblages being similar in the sampling point with high abundance of *O*. *niloticus*, while sampling points with low abundance were dissimilar (Figure 6).

**FIGURE 7:**
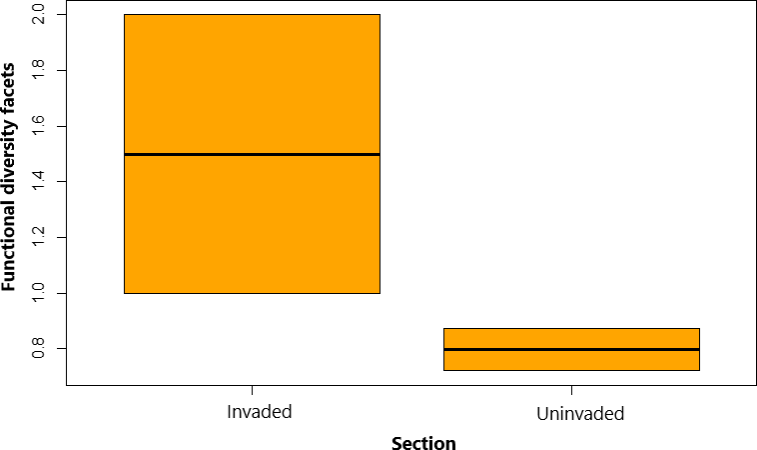
Functional diversity facets comparison between uninvaded and invaded sections of upper Kabompo River (Test 1). Whiskers represent ±*SD*, dashed line in box is median of the dataset, and dash around the box is dataset variation

**FIGURE 8:**
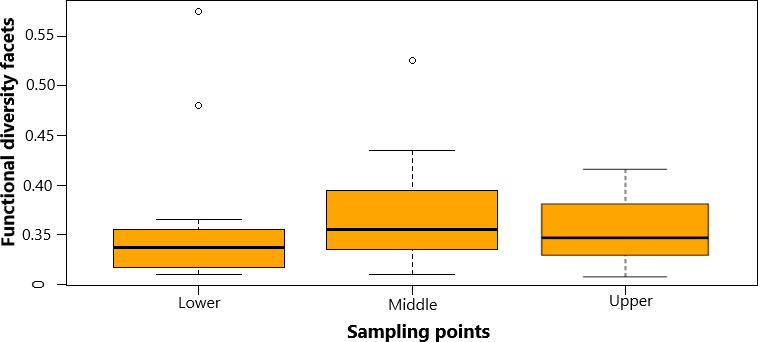
Functional diversity facet comparison summary across sampling points in the invaded section of the upper Kabompo River (Test 2). Whiskers represent ±*SD*, dashed line in the box is median of the dataset, and dash around the box is dataset variation

Functional diversity mean in Test 1, compared between the invaded and the uninvaded sections was significantly different (*p* = 0.002, significant level 0.05). Functional diversity of invaded section was 13% higher compared with the uninvaded section in all the three sampling points (Figure 7). Mean functional diversity values were significantly different (*p* = 4.339, significant level 0.05) observed in the sampling points of the invaded section in Test 2 (Figure 8). We further observed that FD of native species was 9% higher in sampling points that had higher presence of *O*. *niloticus* in 2 of 3 sampling points. The parallel increase in FD in the invaded section observed in this comparison was in 50% of the sampled habitats in the sampling points. Tests 1 and 2, generally showed consistent in most sampling points, suggesting that invasion has considerable influence on the FD in the study area.

Further results of our analysis using the Gowers index on the dissimilarity of traits of native species found that differences in the 9 traits in the sampling points were consistent (Tables 2 and 3). Depending on the sampling points, three traits (body depth, body width, and mouth width) were different among sampling points, but was not significant difference (*p* >.05) between uninvaded and invaded sections in Test 1 (Table 2). The same occurred for native species in the sampling points of the invaded section in Test 2 (Table 3). Functional trait differences observed among native species in the uninvaded section and native species within the sampling points of the invaded section and the average trait values for the section with exotic *O*. *niloticus* were different from those with the absence of it, except for traits related to locomotion (in up to 4 sampling points out of 6 representing 66.7% of the sampling points). Variation in the traits of the native species in sampling points with the presence of *O*. *niloticus* was significantly different across all the species (*p* > 0.05) (in only 2 of 3 sampling points of invaded section, 67%). The three trait values were lower in the invaded section that was with than the one without *O*. *niloticus* (Table 3).

**TABLE 2:** Gower dissimilarity index of the functional trait of the uninvaded section of the upper Kabompo River

Traits	ED	GL	MW	SL	HL	SNL	HED	HD	BD	BW	DFL	CPL	CPD
ED													
GL	0.23												
MW	0.04	0.25											
SL	0.23	0.01	0.19										
HL	0.03	0.14	0.02	0.19									
SNL	0.31	0.03	0.27	0.40	0.27								
HED	0.26	0.14	0.23	0.37	0.22	0.12							
HD	0.26	0.54	0.21	0.31	0.20	0.41	0.37						
BD	0.24	0.15	0.37	0.40	0.37	0.56	0.53	0.22					
BW	0.38	0.03	0.26	0.33	0.27	0.45	0.42	0.27	0.29				
DFL	0.31	0.31	0.50	0.64	0.50	0.26	0.30	0.65	0.75	0.69			
CPL	0.44	0.21	0.41	0.55	0.40	0.17	0.22	0.55	0.67	0.59	0.10		
CPD	0.23	0.21	0.40	0.55	0.40	0.16	0.21	0.53	0.69	0.59	0.09	0.02	

Abbreviations: BD, body diameter; BW, body width; CPD, caudal peduncle depth; CPL, caudal peduncle length; DFL, dorsal fin length; ED, egg diameter; GL, gut length; HD, head depth; HED, head eye diameter; HL, head length; MW, mouth width; SL, standard length; SNL, snout length.

**TABLE 3:** Gower dissimilarity index of the functional trait of the invaded section of the upper Kabompo River

Traits	ED	GL	MW	SL	HL	SNL	HED	HD	BD	BW	DFL	CPL	CPD
ED													
GL	0.15												
MW	0.19	0.02											
SL	0.16	0.04	0.23										
HL	0.37	0.24	0.23	0.27									
SNL	0.34	0.34	0.23	0.03	0.25								
HED	0.22	0.23	0.49	0.29	0.44	0.29							
HD	0.28	0.12	0.46	0.23	0.41	0.24	0.11						
BD	0.28	0.06	0.32	0.23	0.43	0.24	0.31	0.30					
BW	0.36	0.51	0.25	0.25	0.40	0.25	0.47	0.43	0.22				
DFL	0.47	0.17	0.32	0.35	0.47	0.37	0.50	0.50	0.22	0.20			
CPL	0.49	0.11	0.35	0.31	0.37	0.30	0.52	0.49	0.33	0.26	0.35		
CPD	0.35	0.08	0.63	0.41	0.59	0.42	0.16	0.21	0.42	0.61	0.62	0.67	

Abbreviations: BD, body diameter; BW, body width; CPD, caudal peduncle depth; CPL, caudal peduncle length; DFL, dorsal fin length; ED, egg diameter; GL, gut length; HD, head depth; HED, head eye diameter; HL, head length; MW, mouth width; SL, standard length; SNL, snout length.

## 4 DISCUSSION

In this study, it was discovered that the presence of *O*. *niloticus* is unequivocally connected to the functional and taxonomic arrangement of native species in the upper Kabompo River segments. Thus, the result of the study showed that the hypothesis was rejected, because of the observed changes in the TD and FD in the river. It was observed that the invaded section is taxonomically less, but functionally more diverse than uninvaded section, this was clarified further after investigation in Test 2 (Figures 3 and 7). Hence, the use of space for time subtraction method to fully understand invasion processes is not comprehensive. As such, the finding of this study suggested that *O*. *niloticus* occupied taxonomic and functional gaps in the invaded section of the river. Similarly, alien species often occupy unfilled niche of natives, than forming new niche in the communities (Le Roux et al., 2020; Weyl et al., 2020; Zengeya et al., 2020).

Our results in Test 1 showed that the native species in invaded section is taxonomically more diverse than uninvaded section. We could not draw any conclusion whether such increase in dissimilarity among native species was as a result of invasion, as shown by the aforementioned test. However, the findings appear not to be supported by the biotic opposition theory intended for the species extravagance, which expresses that expanded species variety should cause intrusion protections (Ellender et al., 2018; Loiola et al., 2018; van Wilgen et al., 2020; Zengeya et al., 2020). Similarly, this study strongly agreed with the niche‐filling prediction (Jardine et al., 2017; Stuart‐Smith et al., 2013; Thuiller et al., 2010). This prediction simulates that native species in uninvaded section can be related taxonomically to each other than in community that are invaded. Following niche‐filling prediction, it is conceivable that in uninvaded communities, the specialties accessible in a gathering of species are more filled. Opposite to the community that is invaded, the accessible specialties are left open by local species that migrate to other areas, providing an unoccupied space for *O*. *niloticus* to invade (Loiola et al., 2018; Le Roux et al., 2020; Zengeya et al., 2020). The niche space in the ecosystem where available can trigger invasion resulting from the presence of other fishes that are ecologically different (Ellender et al., 2018; Jardine et al., 2017; Stuart‐Smith et al., 2013). This was evident from our study conducted in the sampling points of the invaded section in the second comparison. The more prominent TD and FD could prompt more noteworthy protection from invasion because of filled specialty as predicted before (Ellender et al., 2018; Hill et al., 2020; Zengeya et al., 2020). However, this was not established in our findings, to a great extent on the grounds that the vast majority of the accessible examinations did not survey separating inside given explicit species habitat, for example, the ones provided here. In some sampling points of the invaded section, increase in TD and FD was strongly associated with the absence of *O*. *niloticus*.

Exotic species can possibly take an advantage in filling ecological spaces that are unoccupied. This may be more rapid in communities that are highly taxonomically and functionally diverse. The exotic species in community outside its range fills such spaces successfully in the freshwater ecosystem like observed in our study. The findings showed some taxonomic redundancy in some communities with less abundance of *O*. *niloticus*. Increase in redundancy among native species correlates with a decrease in TD (Devictor et al., 2010; Ellender et al., 2018; Le Roux et al., 2020; Scheffer et al., 2015). This suggested the presence of *O*. *niloticus* in the invaded section is associated with the lower taxonomic available among native species. These impacts are underscored in the taxonomic distance among native species, bringing TD estimations of uninvaded section beneath that of invaded section. In contrast, FD remained higher in the invaded than uninvaded sections. This reliable example across the sampling areas is in concurrence with contrasts in TD and FD detailed by past examinations (Hill et al., 2020; Jardine et al., 2017; Ordonez, 2014; van Wilgen et al., 2020). Our outcomes additionally recommended that the irregularity among TD and FD in invaded versus uninvaded areas was brought about by low systematically monitored fishes in a community. This could suggest that the native species assembly structure was influenced by invasion of *O*. *niloticus* in the upper Kabompo River.

The presence of exotic species is closely associated with fish assemblage structure in community evaluated at different scales (Ellender et al., 2018; Zengeya et al., 2020), indicated that the presence of *O*. *niloticus* is identified with lower TD. Sampling points of the invaded section with higher occurrence of *O*. *niloticus* were found in taxonomically more diverse habitats of native species (van Wilgen Measey & Richardson, 2020; van Wilgen et al., 2020a; Zengeya et al., 2020). Contrary findings were observed from abiotic and biotic influences on taxonomic structure of the native species in the communities (Devictor et al., 2010; Jardine et al., 2017; Lisosova et al., 2015; Loiola et al., 2018; Mayfield & Levine, 2010). This study showed lower invasion in some sampling points due to filtered from the species pools. Environmental filtering is a leading cause of species pool filtering of species within communities (Le Roux et al., 2020; Swenson & Enquist, 2007; Weyl et al., 2020). Broad‐scale community assembly is regarded as being influenced by the environmental filtering hypothesis. Contrary, narrow scale is by far influenced by interspecific interaction than environmental filtering effect (Hill et al., 2020; Li et al., 2015). This suggested that niche filling plays a pivotal role at community scale regarding species assembly.

Although the trait means in the uninvaded section in the first comparison showed weaker pattern (Figure 4), the observed difference in the species trait means in the invaded section was small due to the presence of *O*. *niloticus*. This again suggested that *O*. *niloticus* occupy available niche gaps in section, instead of growing the segment of the communities due to the ecological space occupied by native species. Consequently, dissimilar among the native species were partially compared, particularly on traits related to feeding and locomotion, provided the evidence that we cannot rule out the Darwin's naturalization prediction. In contrast, functional trait dissimilarity change as shown in Tables 3 and 4 was not only related to effects of invasion by *O*. *niloticus*, but also associated with changes in dominant species in sampling points. This was consistent with the findings by Ordonez (2014) and Carboni et al. (2016) which focused on trait mean changes in the functional structure of dominant native species. However, in Test 1 the changes in community trait mean indicated some similarity in invaded than uninvaded sampling points. Therefore, these findings seem to suggest that *O*. *niloticus* may share similar traits with a dominant native species and fosters community coexistence (Le Roux et al., 2020; Schmitz et al., 2015; Wilson et al., 2020). *Oreochromis niloticus* tend to show more aggressiveness in feeding than native species; therefore, the species mean body depth, and mouth width, snout length, and body width increase with invasion in most omnivorous fishes. However, in some habitats that had thick vegetation, the body depth decreased, such as *Oreochromis macrochir*, over *Coptodon rendalli*, *Tilapia spermanii*, *Clarias* species, and *Serranochromis* species due to predominance of these native species. Most sampled points are dominated by exotic species with bigger snout length and more modest caudal peduncle length, giving a more unique feeding and locomotive technique. The study indicated that the traits linked to fast growth rate and fast movement were strongly associated with native species that coexist (Wilson et al., 2020). Furthermore, we observed a difference in snout length between the native congeneric species in the, but not in caudal peduncle measurements as detected in the second comparison. This suggests that feeding traits in invaded sampling points contrasted with uninvaded sampling points were unequivocally affected by the presence of *O*. *niloticus*. Similarly, the findings of the other studies showed similar observation with a similar *O*. *niloticus* invasion pattern (Devictor et al.,l., 2010; Schmitz et al., 2015; Zengeya et al., 2020).

**TABLE 4:** The fish species (*n* = 17) of the upper Kabompo River examined for functional diversity in our study

Scientific Name	Common name	Species code
Alestidae		
*Micralestes acutidens*	Silver robber	1
*Brycinus lateralis*	Striped robber	2
Cichlidae		
*Coptodon rendalli*	Redbreast tilapia	3
*Pseudocrenilabrus philander*	Southern mouthbrooder	4
*Oreochromis niloticus*	Nile tilapia	5
*Sargochromis carlottae*	Rainbow bream	6
*Sargochromis codringtonii*	Dusky bream	7
*Oreochromis macrochir*	Greenhead tilapia	8
*Serranochromis robustus jallae*	Nembwe	9
*Tilapia sparrmanii*	Banded tilapia	10
Clariidae		
*Clariallabes platyprosopos*	Broadhead catfish	11
*Clarias stappersii*	Blotched catfish	12
*Clarias theodorae*	Snake catfish	13
Claroteidae		
*Parauchenoglanis ngamensis*	Zambezi grunter	14
Cyprinidae		
*Enteromius afrovernayi*	Spottail barb	15
*Enteromius barnardi*	Blackback barb	16
*Enteromius eutaenia*	Orangefin barb	17

Finally, this investigation indicates that taxonomic and functional differences of ichthyofauna in communities above and below the natural waterfall could be partially associated with other factors such as different starting communities and hydrological differences than the presence of *O*. *niloticus* sorely. However, in the sampling points of the invaded section should that differences in TD and FD could have resulted from the presence of the *O*. *niloticus*. These changes can adversely affect the assembly cluster of native species and may ultimately affect overall functioning of ecosystems (Finerty et al., 2016; Weyl et al., 2020). Our findings have suggested that within the invaded section *O*. *niloticus* more often occupy unoccupied functional gaps in the upper Kabompo River. This ecological function loss brought about by *O*. *niloticus* as observed by reduction in native species in the invaded section (AES, 2014; DoF, 2018). Moreover, the increased redundancy of native species in the invaded section and also in sampling points that had high dominance of *O*. *niloticus* was as a result of similarity among native species. Different levels of invasion by *O*. *niloticus* documented here have evidently shown that the observed diversity of native species may likely affect the overall ecosystem function in the upper Kabompo River.

To enable us understand functional diversity comprehensively, future studies should be conducted frequently (annually) for effective observation of any advancement in functional traits of the native fishes as a result of the invasion. There is also need to incorporate traits of fish species at individual level and multifaceted approach to better understanding diversity of the upper Kabompo River.

## CONFLICT OF INTEREST

None declared.

## AUTHOR CONTRIBUTIONS

**Arthertone Jere:** Conceptualization (lead); Data curation (lead); Formal analysis (lead); Methodology (lead); Writing‐review & editing (equal). **Wilson Lazaro Jere:** Formal analysis (supporting); Methodology (equal); Supervision (equal); Writing‐review & editing (equal). **Austin Mtethiwa:** Supervision (supporting); Writing‐review & editing (equal). **Daud Kassam:** Supervision (supporting); Writing‐review & editing (equal).

## Data Availability

The data that have been used in this study are available, and Dryad data repository was used to archive the data. The authors cited in this document have acknowledged that data should be stored in the Dryad data repository https://doi.org/10.5061/dryad.dfn2z352f.
